# Enhancement of third-harmonic generation in all-dielectric kite-shaped metasurfaces driven by quasi-bound states in the continuum

**DOI:** 10.1515/nanoph-2024-0194

**Published:** 2024-06-03

**Authors:** Hui-Hsin Hsiao, Jou-Chun Hsieh, Ai-Yin Liu, Kuang-I Lin, Yi-Chien Hsu

**Affiliations:** Department of Engineering Science and Ocean Engineering, 33561 National Taiwan University, Taipei 10617, Taiwan; Institute of Electro-Optical Engineering, 34879 National Taiwan Normal University, Taipei 11677, Taiwan; Graduate Institute of Photonics and Optoelectronics, 33561 National Taiwan University, Taipei, 10617, Taiwan; Core Facility Center, 34912 National Cheng Kung University, Tainan 70101, Taiwan

**Keywords:** quasi-bound states in the continuum, Fano line-shape, all-dielectric metasurfaces, nonlinear optics, third harmonic generation

## Abstract

We develop a new all-dielectric metasurface for designing high quality-factor (*Q*-factor) quasi-bound states in the continuum (quasi-BICs) using asymmetry kite-shaped nanopillar arrays. The *Q*-factors of quasi-BICs follow the quadratic dependence on the geometry asymmetry, and meanwhile their resonant spectral profiles can be readily tuned between Fano and Lorentzian lineshapes through the interplay with the broadband magnetic dipole mode. The third-harmonic signals of quasi-BIC modes exhibit a gain from 43.4- to 634-fold enhancement between samples with an axial-length difference of 15 nm and 75 nm when reducing the numerical aperture of the illuminating objective lenses in nonlinear measurement, which is attributed to the increasing illumination spot size and the less contribution from the large oblique incident light for establishing quasi-BIC modes with high-*Q* spectral profile and strong near-field intensity. The silicon-based metasurfaces with their simple geometry are facile for large-area fabrication and open new possibilities for the optimization of upconversion processes to achieve efficient nonlinear devices.

## 1 Introduction

Dielectric metasurfaces have emerged as a versatile platform for enhancing light–matter interactions at the subwavelength scale and applied for a variety of applications such as sensors [1], [2], [3], [4], [5], [6], nonlinear optics [7], [8], [9], [10], [11], and nanolasers [12], [13], [14]. Compared to their plasmonic counterparts, the displacement currents induced in the interior of the dielectric nanocavity are capable of generating strongly enhanced local fields tightly bound within the nanocavity volume due to their intrinsic low dissipation loss. The associated Mie-type resonances such as electric dipolar (ED) and magnetic dipolar (MD) modes as well as the anapole modes have been widely explored in various individual or periodic dielectric nanostructures for boosting nonlinear effects such as third-hormonic generation (THG) [15], [16], [17] or second-hormonic generation (SHG) [18], [19]. Recently, an eminent group of dielectric metasurfaces supporting Fano resonances [20] and quasi-bound states in the continuum (quasi-BICs) [21] has been developed to achieve high quality-factor (*Q*-factor) resonances by manipulating the coupling strength between the resonant states to further minimize the radiative leakage.

Fano resonance, with a characteristic asymmetric spectral line shape, is known to be arisen from the interference between a discrete state (dark mode) and a continuum of propagation modes (bright mode) [22]. For example, various dielectric coupled systems such as quadrumer nanodisks [23] and the combination of nanodisks and nanobars [24] have been designed to support high *Q*-factors Fano resonances and achieved efficient generation of TH signals. Later, a more generalized concept of BIC modes has been widely studied in photonics, which occurs due to the forbidden coupling between the eigenmodes of resonators and the external propagating modes, thus resulting in a nonradiative localized state embedded in the radiative continuum [21]. In an ideal scenario, the BIC exhibits an infinite *Q*-factor so that renders it unobservable in the spectrum due to its vanishing spectral linewidth. To employ the quasi-BICs, one can introduce slight perturbation into the resonators to allow the leakage of small radiation into free space by either tuning the geometry or excitation parameters [25]. Recently, the transition between Fano resonances (asymmetric line-shape) and a quasi-BIC (symmetric Lorentzian line-shape) have been investigated in isolated AlGaAs nanodisks under structured cylindrical beam excitation [26]. In addition, several works demonstrate the manipulation of Fano interference through the coupling between the localized–localized modes [27], [28], the localized-lattice resonances [29], the lattice-lattice resonances [30], and the localized-quasi-BIC resonances [31]. By tuning the structural geometry or material properties [32], full control of Fano lineshapes as well as the electromagnetically induced transparency (EIT)-like phenomenon can be achieved.

One type of quasi-BICs that manipulate the in-plane symmetry of the composed meta-atoms is known as the symmetry-protected quasi-BICs. To introduce the symmetry breaking into the in-plane geometry, a variety of nanostructures such as tilted nano-ellipse pairs [11], [33], [34], asymmetric nanorod pairs [10], [35], and asymmetric spilt-ring resonator pairs [25], [36] have been demonstrated and showed the characteristic inverse-square law between the structural asymmetry and the *Q*-factors of quasi-BIC resonant linewidths. However, these paired nanostructures usually suffer from severe challenges in fabricating deep subwavelength gaps between the adjacent high aspect-ratio nanoposts and exhibit extra scattering losses due to fabrication imperfection. In addition, other designs are proposed by adding or removing a part of the nanostructure to break the in-plane symmetry, including notched cubes [8], [37], crescent-shaped nanostructures [38], [39], and nanodisks with small broken nanoholes [7], [40]. Nevertheless, the requirement of sharp corners or precisely control of the detuned position for the introduced defects makes it difficult to be accurately fabricated through lithographic techniques, which degrades the performance of the resonators [41].

**Figure 1:**
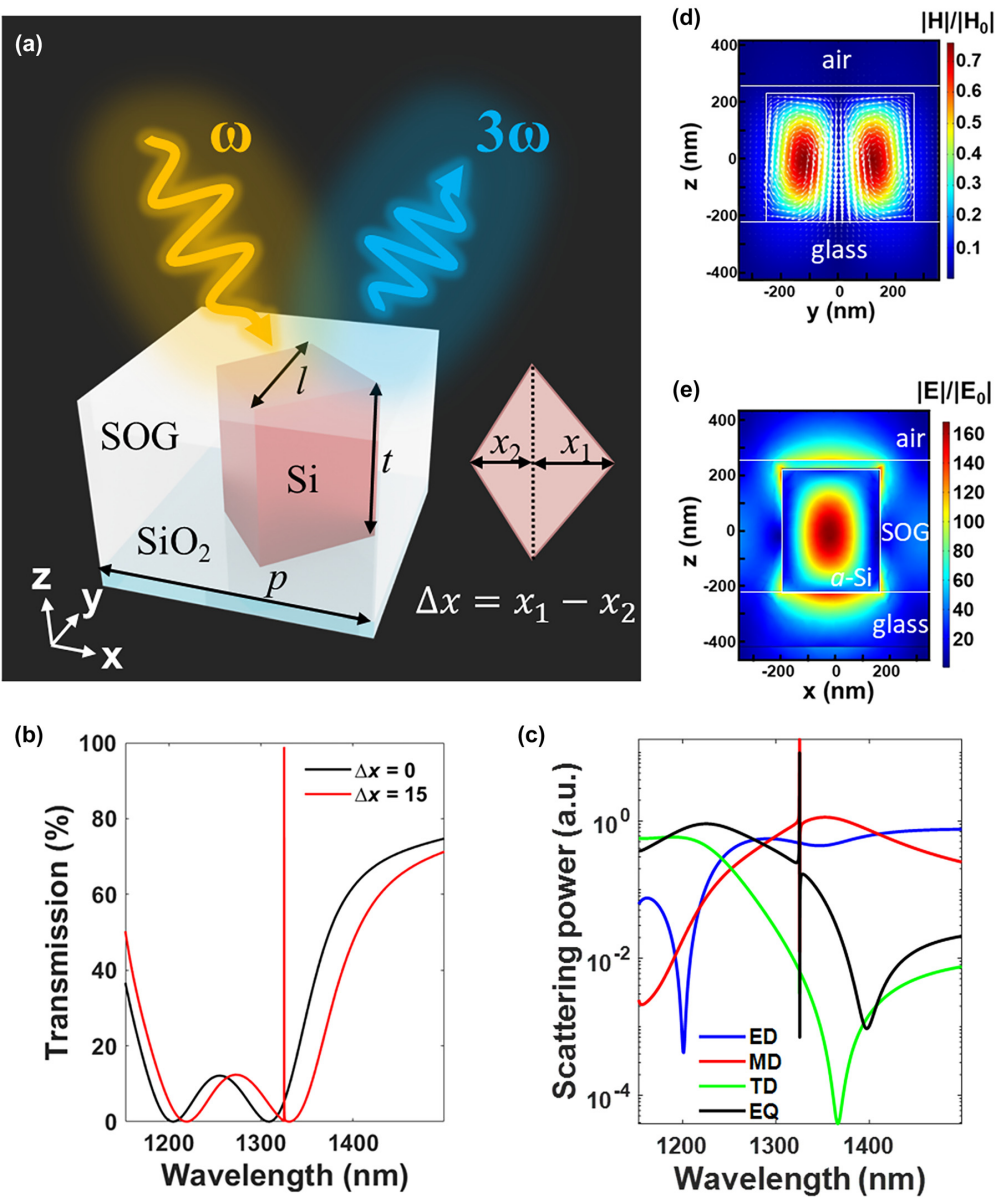
Design of nonlinear all-dielectric metasurfaces. (a) Schematic view of asymmetric kite-shaped metasurfaces. (b) Simulated transmission spectra for kite-shaped nanopillars with symmetric edges (black curve) and an asymmetry level of Δ*x* = 15 nm (red curve). (c) The calculated scattering power of each individual electromagnetic multipole moment for asymmetric kite-shaped nanopillars with Δ*x* = 15 nm. The four strongest multipoles including electric dipole (ED), magnetic dipole (MD), toroidal dipole (TD), and electric quadruple (EQ) are displayed. (d) The magnetic and (e) electric field distributions for Δ*x* = 15 nm at the quasi-BIC resonance (*λ* = 1,325 nm). White arrows in (d) refer to the orientation of displacement currents.

In this work, we have developed a new all-dielectric metasurface for creating quasi-BIC resonances using kite-shaped nanopillar arrays with two asymmetry halves (Figure 1a). The high-*Q* quasi-BIC mode exhibits a dominant MD resonant feature with an extremely large electric-field intensity confined within the nanocavities and manifests itself as a Fano lineshape residing in a broadband MD mode in the spectra. A systematic study was performed to investigate the dependence of quasi-BICs on geometry, the oblique incident angle, as well as the interplay with the broadband MD resonances in affecting the resonant bandwidths and lineshapes of the quasi-BICs. Then, we fabricated a series of kite-shaped nanopillars with different asymmetry parameters and analyze how the THG efficiency depends on the degree of asymmetry and the illuminating condition in nonlinear measurements. Such Si-based designs are compatible with state-of-art complementary metal-oxide-semiconductor technology and sustain large breakdown threshold to the laser power. Meanwhile, the substantial degrees of freedom for substrate materials are beneficial for the on-chip integration.

## 2 Results and discussions

Figure 1a illustrates the unit cell of amorphous Si (*a*-Si) kite-shaped nanopillars designed on a glass substrate. The kite-shaped nanostructure has a fixed length (*l*) of 540 nm along the *y*-direction, a pillar height (*t*) of 450 nm, and is arranged in a square periodicity (*P*) of 700 nm. The length from the center to the right and left edge of the kite shape is defined as *x*_1_ and *x*_2_, respectively. To break the in-plane symmetry, we kept *x*_2_ at 170 nm and varied *x*_1_, thus rendering the asymmetry level decided by Δ*x* = *x*_1_ − *x*_2_. An spin-on-glass layer with a thickness of 480 nm was added to fully cover the dielectric nanopillar arrays. All the electrodynamic simulations were performed by using COMSOL Multiphysics software (COMSOL Inc.), and the charge-current multipole expansion was implemented to analyze the multipolar contributions to the far-field scattering (see computational details in Supplementary Material). Upon normal incidence of *x*-polarized light, Figure 1b displays the simulated transmission spectra for both of the unperturbed (Δ*x* = 0) and perturbed structures with Δ*x* = 15 nm. The discussion of the optical property for the unperturbed structure can be found in Section S2 in Supplementary Material. For both the unperturbed and perturbed structures, two resonant dips occur at the wavelength (*λ*) of 1,220 nm and 1,335 nm, respectively, referring to the electric quadrupole (EQ) and the magnetic dipole (MD) modes according to the multipolar decomposition analysis (Figure 1c). In addition, one additional sharp resonance was excited at *λ* = 1,325 nm for the asymmetric kite-shaped nanostructure (Δ*x* = 15 nm). This symmetry-protected quasi-BIC mode exhibits a dominant MD resonant feature (Figure 1c) by manifesting itself with two circular displacement current loops localized within nanopillars as shown in Figure 1d. Meanwhile, this ultrahigh-*Q* quasi-BIC mode also exhibits an extremely large electric-field enhancement concentrated inside the nanocavity (Figure 1e), which is especially beneficial for nonlinear conversion.

**Figure 2:**
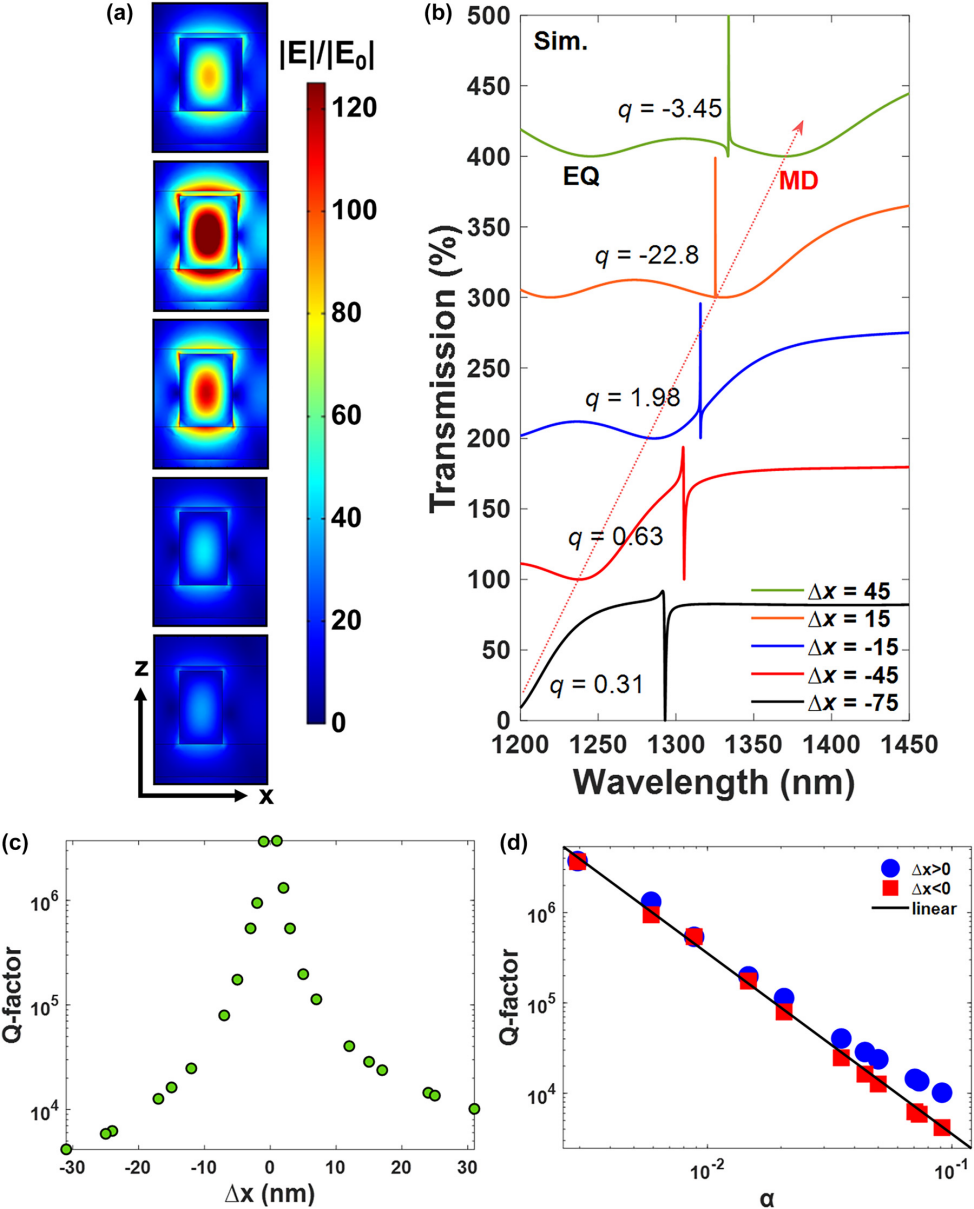
Linear optical properties of asymmetric kite-shaped metasurfaces. (a) Simulated electric-field distributions at quasi-BICs and (b) transmission spectra for kite-shaped nanopillar arrays under different asymmetric levels of Δ*x*. The red-dashed arrow guides to the eye for indicating the broadband MD mode. (c) The dependence of *Q*-factors for quasi-BICs on the asymmetry level of kite-shaped nanopillars. (d) Simulated (dots) and theoretical (black line) *Q* factors of quasi-BIC modes as a function of the asymmetry parameter for kite-shaped nanopillars.

Figure 2b shows the simulated transmission spectra for a series of samples with different asymmetric levels (Δ*x*). As Δ*x* increases, both the high-*Q* quasi-BICs and the broadband EQ and MD modes reshift, while the broadband EQ and MD modes perform a more significant spectral shift compared to that of the quasi-BIC mode. Thus, the quasi-BIC mode is switched from the right-hand side of the MD mode (Δ*x* = −75 nm) to the left shoulder of the MD mode (Δ*x* = 45 nm), rendering a distinct Fano resonant lineshape according to the frequency detuning between the quasi-BICs and the MD mode. To precisely describe a Fano resonant spectral feature, the famous Fano formula is commonly used for the spectral fitting and has the form of [32]

(1) $$\sigma_{\text{Fano}}\left(\delta \right)=|t_{D}{|}^{2}\frac{{\left(q+\Omega \right)}^{2}}{1+\Omega^{2}}$$

where *q* is the Fano parameter, *t*_*D*_ is the nonresonant transfer amplitude via the continuum, *δ* = 2(*ω* − *ω*_0_)/*γ*_0_ is the normalized frequency offset, *ω*_0_ is the frequency of the narrow resonance, and *γ*_0_ is its inverse lifetime. The Fano parameter *q* is used to describe the coupling strength between the fundamental resonances and determines the spectral line shape. As shown in Figure 2b, the calculated *q* values are positive values when the quasi-BIC mode is resided at the right-hand side of the MD resonance (i.e., Δ*x* < 0). As Δ*x* increases, the frequency detuning between the quasi-BICs and the MD mode decreases and gives rise to a Fano lineshape with an increasing *q* value. When Δ*x* = 15 nm, an EIT-like window appears and transforms into a symmetric Lorentzian lineshape with *q* = −22.8. This EIT-like phenomenon is due to the Fano interference between the low-*Q* MD mode and the ultrahigh-*Q* quasi-BIC with zero detuning. When Δ*x* increases to 45 nm, the quasi-BIC mode further shifts to the left shoulder of the MD mode and results in a negative *q* value of −3.45. The tuning of *q* can also be observed when the nanopillar height is changed, and the effects of other geometric parameters on the interplay between the quasi-BIC mode and the broadband MD modes are discussed in Section S3 in Supplementary Material.

In addition, the *Q*-factors of quasi-BICs were also estimated by using a Fano line shape equation to fit the transmission spectra [27],

(2) $$T_{\text{Fano}}={\left|a_{1}+ia_{2}+\frac{b}{\omega -\omega_{0}+i\gamma_{\text{tot}}}\right|}^{2}$$

where *a*_1_, *a*_2_, and *b* are real numbers, *ω*_0_ is the central oscillation frequency, and *γ*_tot_ is the total damping loss. The extracted total *Q* factors (*Q*_tot_) is equal to radiative *Q* factors (*Q*_rad_) since the ohmic loss is excluded (*γ*_ohm_ = 0) in our simulations (i.e., *Q*_tot_ *=* *ω*_0_/2*γ*_tot_ *=* *ω*_0_/2(*γ*_rad_ + *γ*_ohm_) = *Q*_rad_). The estimated *Q*-factors of quasi-BICs from the simulations reveal the expected trend with a significant increasing value for smaller Δ*x* and reaches a value beyond 10^6^ when Δ*x* is close to 0 (Figure 2c). In addition, we used Δ*S*/*S* to define the asymmetry parameter *α*, where *S* is the area of the symmetric nanokite, and Δ*S* is the area of the removing or adding part. As shown in Figure 2d, the calculated *Q*-factors follow the quadratic dependence on the asymmetry parameter very well (i.e., *Q* ∝ (Δ*S*/S)^−2^ = *α* ^−2^). It is interesting to notice that even though the *Q*-factors are theoretically depended on the asymmetry parameter only, the calculated *Q*-factor is generally larger for the cases with Δ*x* > 0 compared to that of Δ*x* < 0 under the same value of $\left|\Delta x\right|$ . Similar trend can be observed from the electric-field distribution of each sample at the quasi-BIC resonance as displayed in Figure 2a. Both the near-field intensities of Δ*x* = ±15 nm show more than two order of magnitude enhancement, and the largest near-field intensity is occurred at Δ*x* = 15 nm with a value beyond 160 in our simulations. For the case with a larger asymmetric parameter, the near-field distribution degrades significantly.

**Figure 3:**
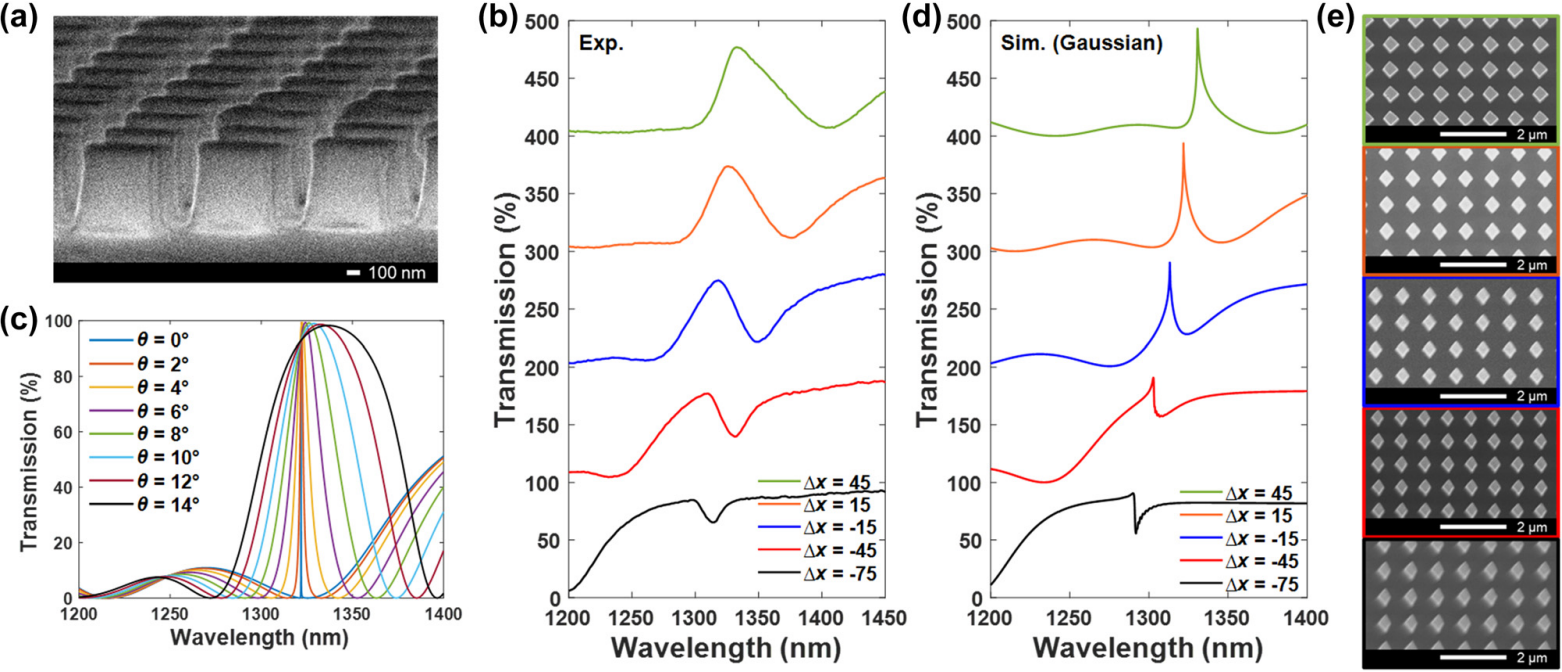
Experimental and simulated transmission spectra of the samples. (a) SEM image of the fabricated sample at the cross-sectional view. (b) Measured transmission spectra for asymmetric kite-shaped nanopillars with different Δ*x*, and (d) the corresponding transmission spectra calculated by summing the simulated spectra under different oblique incident angles in a Gaussian distribution. (c) Simulated transmission spectra for asymmetric kite-shaped nanopillars with Δ*x* = 15 nm under different oblique incidence angles. (e) SEM images of the fabricated samples with Δ*x* = 45 nm (green), 15 nm (orange), −15 nm (blue), −45 nm (red), and −75 nm (black), respectively.

Next, a series of samples were fabricated by standard electron-beam lithography (EBL) followed by reactive ion etching (RIE) process (see fabrication details in Section S4 of Supplementary Material). Figure 3e shows the top-view scanning electron microscopic (SEM) images of the fabricated samples for Δ*x* = −75 nm to 45 nm, respectively, and an oblique SEM image with 90° tilted angle was performed to examine the lateral cross section of nanokites (Figure 3a). The transmission spectra were characterized by an in-housed built microscope equipped with a near-infrared spectrometer (see measurement details in Section S5 of Supplementary Material). As shown in Figure 3b, the measured spectra of the quasi-BIC modes exhibit a redshift trend for larger Δ*x*. Meanwhile, one can observe the resonant linewidths of the quasi-BIC modes are broadening in experiments compared to numerical results (Figure 2b). Different from several previous studies that demonstrates the inevitable surface roughness and dimension variation for the fabricated samples are the dominant effects for the broadening of the resonances [33], we found that the broadening of the quasi-BICs in our design is predominantly attributed to its spectral dependence on the oblique incident angle (*θ*) as displayed in Figure 3c, while the additional material or scattering losses due to fabrication imperfection have a relatively subtle effect to the spectral width (see details in Section S6 of Supplementary Material). Thus, we utilized a Gaussian distribution to estimate the contributions from each incident angle according to the numerical aperture (NA) of the objective lens used in our measurement setup and calculated the transmission spectra as a linear combination of the spectra shone by different incident angles (see details in Section S7 of Supplementary Material). As shown in Figure 3d, the weighted transmission spectra display a shallower resonant depth with a broader resonant linewidth for the quasi-BICs, which fit very well to the experimental results. In addition, we found the quasi-BICs for samples with different Δ*x* exhibit distinct amounts of spectral broadening at oblique incidence, where a severer *Q*-factor degradation was found for the quasi-BIC mode in asymmetric nanokites with a larger cross-sectional area under the same oblique incident angle (Figure S4 in Supplementary Material). Thus, the dependence of the inverse quadratic relationship for quasi-BICs on the asymmetric parameter is hidden in the measured spectra due to the weighted effect.

**Figure 4:**
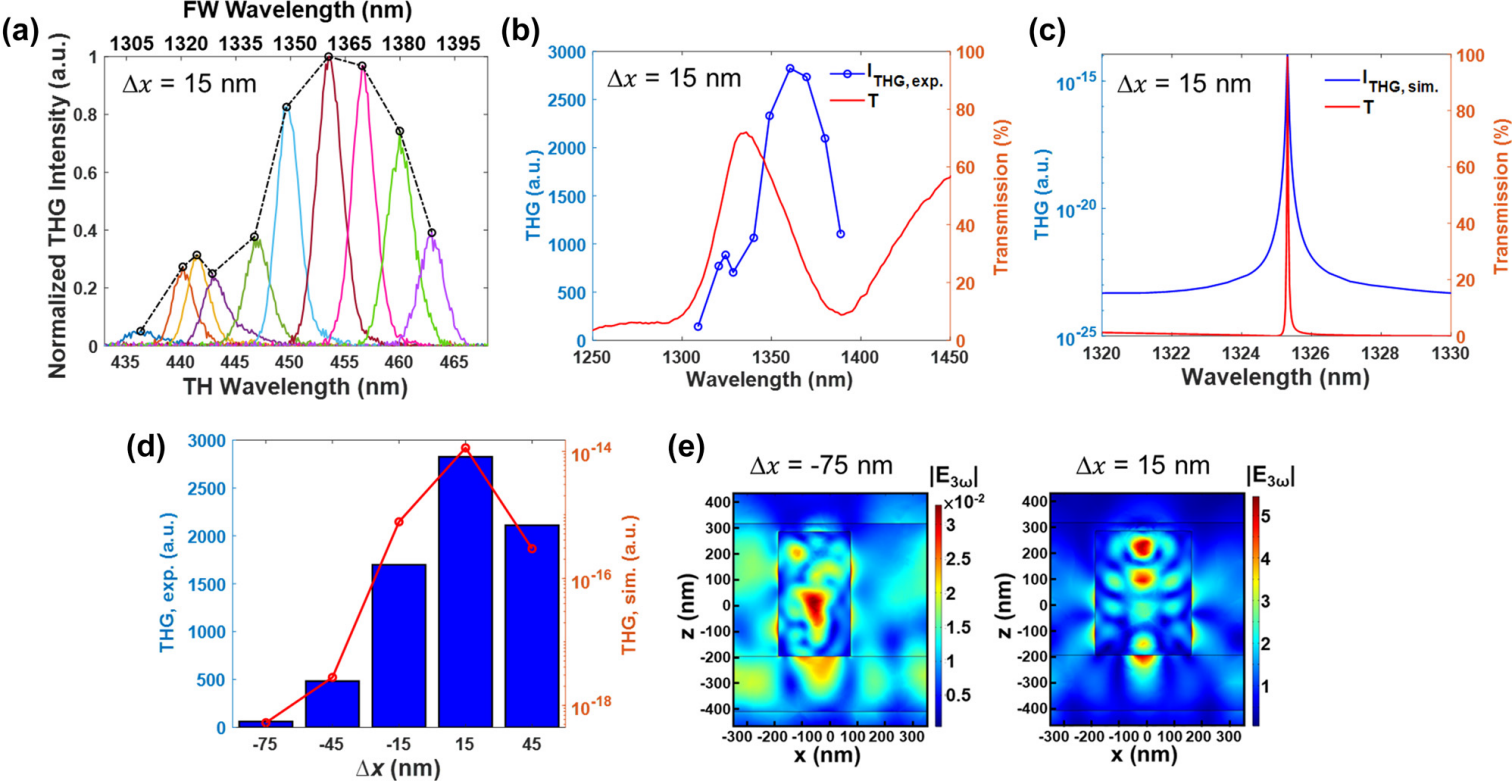
Nonlinear optical properties of asymmetric kite-shaped metasurfaces. (a) Measured THG spectra under different laser wavelength with a fixed initial input power of 100 mW. Color curves refer to the THG signal profile under different fundamental excitation wavelengths, and the black dashed curve represents the envelope of the color curves. (b) Measured and (c) simulated transmission (red curve) and THG (blue curve) spectra for the sample with the minimal asymmetry level of 15 nm. (d) Comparison of the peak intensities for the measured (blue column) and simulated (red curve) THG signals for samples with Δ*x* varied from −75 nm to 45 nm. The objective lens used in THG measurement has a NA value of 0.8. (e) The cross-sectional (*x*–*z* plane) electric-field distributions at the TH wavelength for Δ*x* = −75 nm (left) and 15 nm (right), respectively.

Finally, the THG intensity of *a*-Si nanopillar arrays were measured by an in-housed built multiphoton excitation microscope combined with an optical parametric oscillator (OPO) pumped by a mode-locked Ti: sapphire as the laser source. The laser pulse was focused onto the sample with a spot size of 2 μm in diameter by using a 20× objective with a NA of 0.8. The reflected THG intensity was captured through four photomultiplier tubes (see THG measurement in Section S5 in Supplementary Material). As shown in Figure 4a, the excitation wavelength of the input laser beam was varied to measure the THG response around the quasi-BIC mode for each sample with a sampling wavelength of 5–10 nm. As the input power of the laser beam is calibrated to remain the same magnitude at each fundamental excitation wavelength, the value of TH signals can be compared directly. Figure 4b shows the measured linear spectra and its corresponding TH signal for the sample with Δ*x* = 15 nm. It is worth mentioning that the experimentally observed THG peak exhibits a redshift with respect to the measured transmission peak, while only a slight spectral shift is observed between the simulated transmission and THG spectra as shown in Figure 4c (see computational details in Section S8 of Supplementary Material). Such phenomena may be attributed to the fact that the NA value of the incident objective lens in THG experiments (NA = 0.8) is much larger than the one used during the linear spectral measurements (NA = 0.25), thus leading to a much smaller illumination area and a wider range of oblique incident angles for nonlinear measurements. It should be notable that the size of illumination area will influence the excitation of the quasi-BIC mode as the quasi-BIC mode requires a sufficient number of meta-atoms to sustain their intrinsically collective resonant property [42]. Meanwhile, as the spectral profile and the near-field feature of quasi-BICs in kite-shaped nanopillars strongly depend on the oblique incident angle (Figure 3c), the increasing oblique illuminating components in nonlinear measurements may inevitably cause a more serious spectral shift and broadening effect for THG response. In addition, the intrinsic dispersion of the bulk *χ* ^(3)^ for silicon may also lead to the spectral shift of THG intensity in the vicinity of resonant modes.

**Figure 5:**
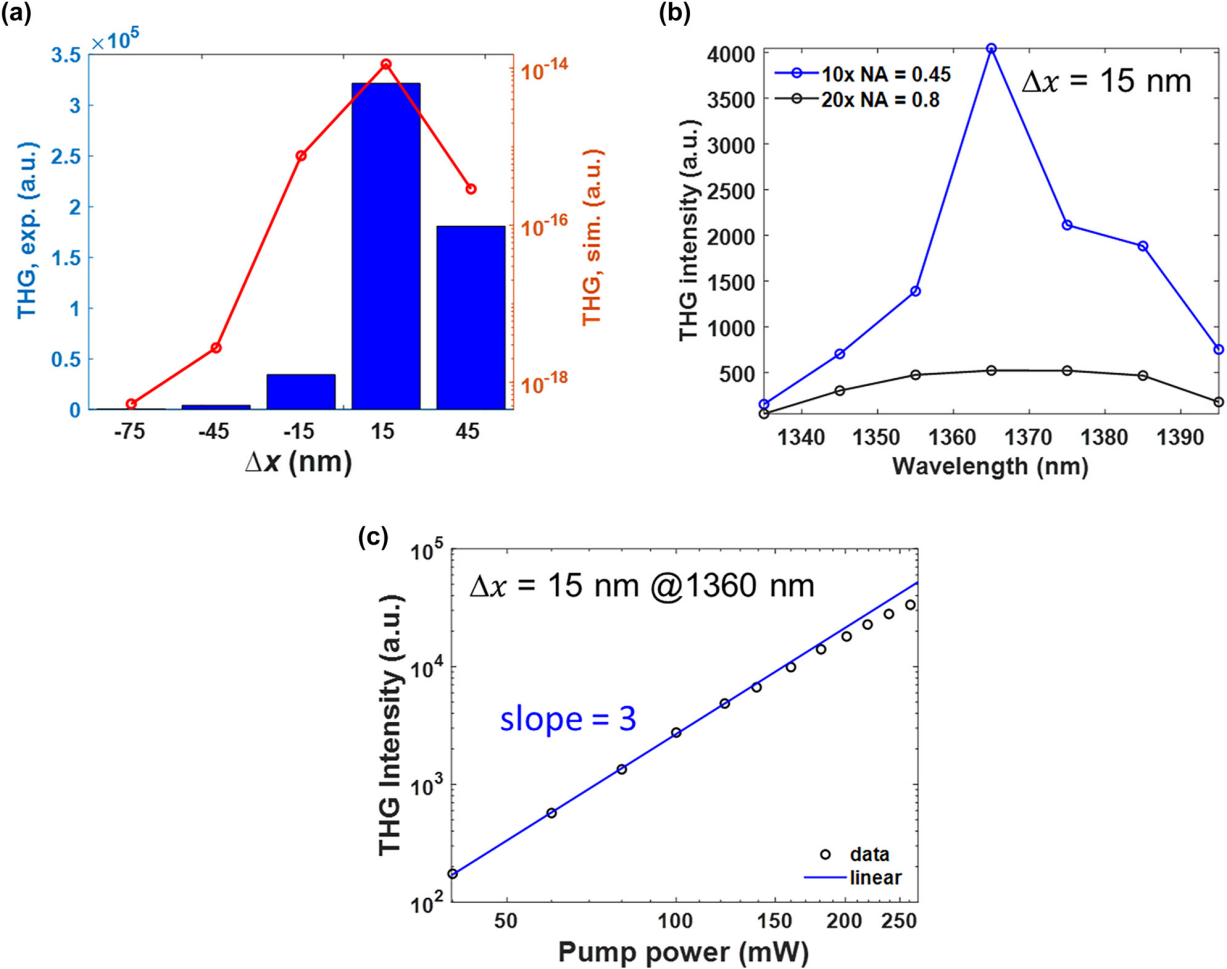
Effects of the objective lens in nonlinear measurements. (a) Comparison of the peak intensities for the measured (blue column) and simulated (red curve) THG signals for samples with Δ*x* varied from −75 nm to 45 nm. The objective lens used in THG measurement has a NA value of 0.45. (b) THG intensities for Δ*x* = 15 nm measured by a 20× objective lens with NA = 0.8 (black curve) and a 10× objective lens with NA = 0.45 (blue curve) under the same input laser power. (c) The log–log plot for Δ*x* = 15 nm under a pump wavelength of 1,360 nm. The blue line is the fitting data with a third-order power dependence.

Figure 4d summarizes the measured and simulated peak value of the THG intensity for samples with different asymmetry levels. Both the measured and simulation results show that the highest THG intensity occurs at Δ*x* = 15 nm and starts to decrease for samples with larger asymmetry levels. This demonstrates that even though the collected THG signals suffer from the similar weighted effect as in the linear measurement, the strongest induced field for the high-*Q* quasi-BIC modes under normal incidence still performs a predominant contribution to the measured nonlinear signals owing to the cubic dependence of THG on the electric field. The simulated THG response for Δ*x* = 15 nm achieves four order of magnitude enhancement compared to that of Δ*x* = −75 nm. Such giant enhancement can also be observed from the calculated nonlinear electric-field distributions at the TH wavelength ( $\left|E\left(3\omega \right)\right|$ ). As shown in Figure 4e, the $\left|E\left(3\omega \right)\right|$ of Δ*x* = 15 nm exhibits beyond two-order of magnitude stronger than that of Δ*x* = −75 nm. On the other hand, the experimental results show the THG signal of Δ*x* = 15 nm reaches 46.4 times enhancement in comparison to that of Δ*x* = 75 nm and is 2,900-fold larger than that of an un-patterned Si film (the THG enhancement for all the samples are summarized in Table 1 of Supplementary Material). To further examine the effect of the NA value for the incident objective lens in nonlinear measurements, we replaced the objective lens to a lower NA value of 0.45, corresponding to an estimated focal spot diameter of 3.7 μm. As shown in Figure 5a, a more significant variation of the measured peak THG intensity can be observed among these samples, and the THG signals between the samples with Δ*x* = 15 nm and −75 nm increases to 643 times enhancement. Figure 5b compares the measured THG intensities for the sample with Δ*x* = 15 nm illuminating by using the two different NA objective lenses under the same input laser power. The obtained signal is 7.7 times larger for the objective lens of NA = 0.45 than that acquired by using the objective lens of NA = 0.8. This gives us an evidence that the quasi-BIC mode is more completely established to sustain a strong localized field in the nanocavities when the number of the array elements shone by the laser light increases and results in a more predominant nonlinear effects. In addition, the decrement of the contributions from oblique incident light is also beneficial to maintain the high-*Q* performance of quasi-BIC modes and achieve larger THG enhancement.

Finally, the dependence of the measured output power on the pump power for the metasurfaces was investigated. Figure 5c displays the measured THG under different initial excitation power varied from 40 to 260 mW at *λ* = 1,360 nm for Δ*x* = 15 nm. For the average input powers below 140 mW, the THG power follows the expected trend of a cubic dependence on the FW excitation power (Figure 5c). For higher pump intensities, the dependence shows a typical saturation behavior, which may arise from the laser heating effect that causes a small refractive-index change of *a*-Si resonators and leads to the resonant wavelength shift [16], [43], [44]. The absolute THG conversion efficiency defined as *η*_TH_ = *P*_TH_/*P*_in_, where *P*_TH_ is the collected THG emission power and *P*_in_ is the input pump power, was estimated to be 1.75 × 10^−6^ at the average pump power of 4.23 mW for Δ*x* = 15 nm (peak pump intensity of 8.2 GW/cm^2^). The peak power intensity was calculated based on the temporal pulse width, the repetition rate, and the spot size of the fundamental beam. Since *η*_TH_ increases quadratically with the pump power [16], the normalized conversion efficiency defined by $\xi_{\text{TH}}=P_{\text{peak}\;-\;\text{TH}}/P_{\text{peak}\;-\;\text{in}}^{3}$ (*P*_peak−TH_ is the peak TH power; *P*_peak−in_ is the peak pump power) [45] was also evaluated under the assumption that the pulse duration time of THG signal is the same as the pump laser and yields a value of 2.63 × 10^−11^ W^−2^.

## 3 Summary and outlook

In conclusion, we designed an all-dielectric kite-shaped metasurface driven by quasi-BICs and demonstrated it as an efficient optical platform for boosting the THG signals. By manipulating the in-plane geometry of the kite-shaped meta-atoms, the symmetry-protected BIC was transformed into an accessible quasi-BIC with a tunable *Q*-factor and enhanced near-fields confined in the interior of the dielectric nanocavity. Meanwhile, tuning of *q* for the Fano spectral profile of quasi-BIC modes is achieved due to the interplay between the quasi-BICs and the broadband MD mode. Such quasi-BIC mode was found to show strong dependence on the oblique incident angle, resulting in a predominant redshifted and broadening spectral profile at larger incident angle. The well-tuned *Q*-factor and the near-field enhancement of quasi-BIC modes renders four order of magnitude THG enhancement in simulations when comparing the nonlinear signals between the kite-shaped metasurfaces with Δ*x* = 15 nm and Δ*x* = −75 nm, while their measured THG signals exhibit 46.4-fold and 634-fold enhancement when the incident objective lenses have the NA values of 0.8 and 0.45, respectively. The obtained THG signal for Δ*x* = 15 nm increases 7.7 times for NA = 0.45 in comparison to the result measured by NA = 0.8. This is attributed to the increasing illumination spot size for establishing quasi-BIC modes due to their collective antenna array effect. Meanwhile, the obtained signal with a less contribution from the large oblique incident light has a mild degradation to the *Q*-factor and the near-field intensity of quasi-BICs. Our findings open a novel avenue for developing quasi-BIC driven all-dielectric metasurfaces for boosting the TH upconversion efficiencies.

## Supplementary Material

Details of simulations, sample fabrication, and linear and nonlinear optical characterization; optical property of symmetric kite-shaped nanopillars; geometric dependence of quasi-BICs; effects of fabrication imperfections and oblique incident angles on quasi-BICs; nonlinear numerical model for the THG response of all-dielectric metasurfaces; THG enhancement for nanokites with different asymmetry levels.

## Supplementary Material

Supplementary Material Details
