# Supplementary material for: Enhancement of third-harmonic generation in all-dielectric kite-shaped metasurfaces driven by quasi-bound states in the continuum
Source: Nanophotonics. 2024 Jun 3;13(17):3155–64. doi: 10.1515/nanoph-2024-0194 (PMC11501343; doi:10.1515/nanoph-2024-0194)
Supplement: Supplementary file 1 — Supplementary Material Details [file j_nanoph-2024-0194_suppl_001.pdf]

Supplementary Material

Enhancement of Third-Harmonic Generation in all-Dielectric Kite-shaped Metasurfaces driven by Quasi-bound States in the Continuum

Hui-Hsin Hsiao[1,*], Jou-Chun Hsieh[2], Ai-Yin Liu[3], Kuang-I Lin[4], and Yi-Chien Hsu[2]

[1]Department of Engineering Science and Ocean Engineering, National Taiwan University, Taipei 10617, Taiwan

[2]Institute of Electro-Optical Engineering, National Taiwan Normal University, Taipei 11677, Taiwan

[3]Graduate Institute of Photonics and Optoelectronics and Department of Electrical Engineering, National Taiwan University, Taipei, 10617, Taiwan

[4]Core Facility Center, National Cheng Kung University, Tainan 70101, Taiwan

*E-mail: hhhsiao@ntu.edu.tw.

Section S1: Simulations

The electromagnetic numerical simulations were conducted utilizing the finite element method within the COMSOL Multiphysics software, operating in the frequency domain to evaluate both linear and nonlinear electromagnetic responses. Periodic boundary conditions were implemented on four sides of the unit cell to simulate the square lattice. The refractive indices of the glass substrate, SOG, and *a*-Si were set to be 1.5, 1.45, and 3.5, respectively. The incident plane wave was normal incidence with *x*-polarization. In addition, a multipole decomposition method was employed to analyze the multipolar electromagnetic fields contributing to far-field scattering by integrating the induced current over the structures [1],

$$\vec{p} = \frac{1}{i\omega}\int \vec{J}\, d^3r$$

$$\vec{m} = \frac{1}{2c}\int \vec{r}\times\vec{J}\ \ d^3r$$

$$\vec{T} = \frac{1}{10c}\int [(\vec{r}\cdot\vec{J}\,) - 2r^2\vec{J}]\, d^3r$$

$$\vec{Q}_{\alpha\beta}^{(e)} = \frac{1}{2i\omega}\int [r_\alpha J_\beta + r_\beta J_\alpha - \frac{2}{3}(\vec{r}\cdot\vec{j}\,)\delta_{\alpha\beta}]\, d^3r$$

$$\vec{Q}_{\alpha\beta}^{(m)} = \frac{1}{3c}\int [(\vec{r}\times\vec{J}\,)_\alpha r_\beta + (\vec{r}\times\vec{J}\,)_\beta r_\alpha]\ \ d^3r$$

where $\vec{p}$, $\vec{m}, \vec{T}, \vec{Q}_{\alpha\beta}^{(e)}$, and $\vec{Q}_{\alpha\beta}^{(m)}$ denote as electric dipole, magnetic dipole, toroidal dipole, electric quadruple, and magnetic quadruple moments, respectively. *r* represents the distance vector from its origin at the center of nanopillar to the point position in a Cartesian coordinate. α, β denote *x*, *y*, *z*, and *c* is the speed of light in a vacuum.

Section S2: Optical property of symmetric kite-shaped nanopillars

Figure S1 shows the simulated and measured spectra of the symmetry kite-shaped nanopillars ($\Delta x = 0$). Upon the illumination of *x*-polarized light, one can observe two resonant dips around λ = 1200 nm and 1310 nm, referring to the electric quadrupole (EQ) and the magnetic dipole (MD) modes, respectively. Without the breaking of in-plane symmetry, the BIC mode is unobservable in the spectrum. The measured spectrum (blue curve in Figure S1b) agrees well with the simulation result, while the EQ and MD modes are slightly merged in the experimental result. On the other hand, under the incidence of *y*-polarized light, the resonant dips around λ = 1200 nm and 1400 nm also belong to the EQ and MD resonances, respectively, according to the multipolar decompositon analysis, while the resonant peak at λ = 1130 nm is attributed to the electric dipole resonance. These resonant features can be observed in the measured spectra for *y*-polarized light (red curve in Figure S1b).

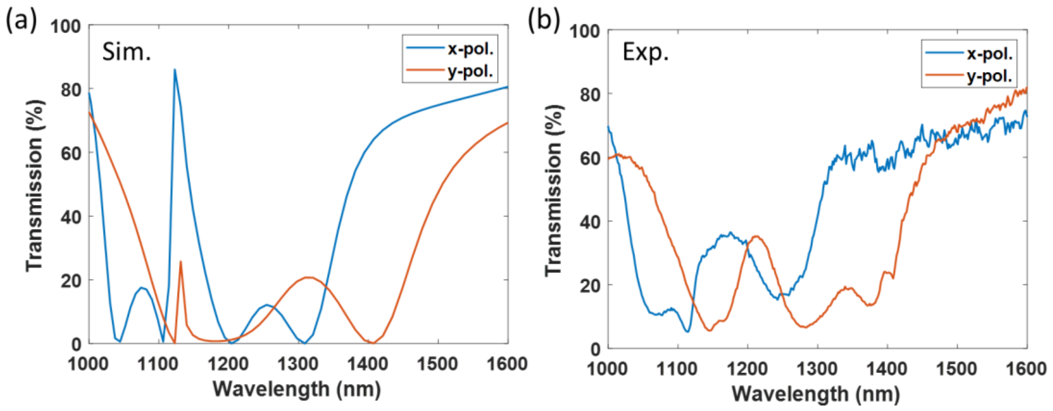

Figure S1. (a) Simulated and (b) measured transmission spectra for symmetry kite-shaped nanopillars under the incidence of *x*- (blue) and *y*-polarized light (red), respectively.

Section S3: Geometric dependence of quasi-BICs

We vary the periodicity (*p*), the thickness (*t*), and the axis length perpendicular to the light polarization (*l*) for the kite-shaped nanopillars to investigate the geometric dependence of quasi-BICs. Generally, when these geometric parameters increase, both the quasi-BIC and the broadband MD resonance redshift, but they also perform a slightly different amount of the spectral shift depended on each parameter as shown in Figure S2. This property enables us to control the frequency detuning between the high-Q quasi-BIC and the broadband MD resonance, resulting in a varation of Fano lineshapes as well as the electromagnetically induced transparency (EIT)-like phenomenon.

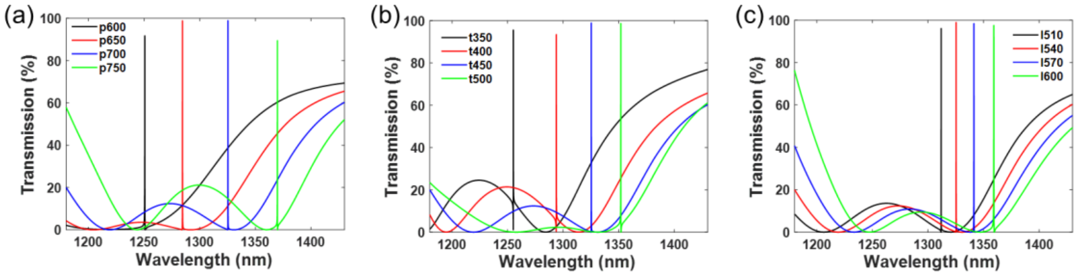

Figure S2. Simulated transmission spectra under the variation of (a) the periodicity, (b) the thickness, and (c) the axis length perpendicular to the light polarization for kite-shaped metasurfaces with Δx = 15 nm.

Section S4: Fabrication

A 450-nm-thick *a*-Si film was first deposited on a glass substrate by plasma-enhanced chemical vapor deposition (Oxford PlasmaPro system 100). Then, a layer of electron-beam resist (ZEP520A:ZEPA = 1:1) was spin-coated onto the *a*-Si film and followed by the spin-coating of an Espacer layer to prevent the charging effect during the electron beam lithography (EBL) process. The patterns were defined using an EBL system (ELS-7000) operating at an acceleration voltage of 100 kV and a beam current of 30 pA. The exposure area of each pattern was 100×100 μm^2. After the exposure, the sample underwent rinsing with deionized water to remove the Espacer layer and was then developed in a ZED-N50 solution. Next, a 30-nm-thick chromium film was deposited via electron-beam evaporation, followed by a lift-off process utilizing N,N-dimethylacetamide (ZDMAC) to transfer the patterns onto chromium disks, serving as hard masks for the subsequent reactive ion etching (RIE) process. The RIE process employed a reaction gas mixture of CF_4 and O_2 at an RF power of 75 W (SAMCO RIE-10NR), resulting in the formation of nanopillars. Subsequently, the chromium disks were removed using a chromium etchant, leaving behind the desired nanopillar structures. Finally, a layer of spin-on-glass (SOG) was spin-coated onto the fabricated nanopillars to complete the process.

Section S5: Optical characterization

Linear optical measurements

The optical properties of samples were characterized by an in-housed-built microscope (10× Infinity Corrected objective, NA = 0.25) equipped with a near-infrared spectrometer (Ocean optics, InGaAs detector). A halogen lamp was used as the light source to impinge the sample with a focal spot size of approximately 100 μm. A CCD camera was incorporated to the system to capture the location of the fabricated pattern, and the spectra were measured by the spectrometer.

Nonlinear optical measurements

The third harmonic generation measurement was performed by using an in-housed built multiphoton excitation microscope [2]. An optical parametric oscillator combined with a mode-locked Ti: sapphire laser (Coherent Chameleon Vision II) is employed to produce a tunable excitation light source with a wavelength range from 680 nm to1600 nm. The laser pulse width is 140 to 200 fs, and the repetition rate is 80 MHz. The laser pulse was focused onto the sample by using (1) 20 × objective with a numerical aperture of 0.8, corresponding to a spot size of 2 μm in diameter and (2) 10 × objective with a numerical aperture of 0.45, corresponding to a spot size of 3.7 μm in diameter. The backward TH signal was detected using four photomultiplier tubes accompanied with band-pass filters to acquire signals in the wavelength of interest.

Section S6: Effects of fabrication imperfections on quasi-BICs

To account for non-radiative loss mechanisms in the fabricated samples, such as defects in deposited films, surface roughness, and misalignments, the extinction coefficient (k) is artificially increased to values orders of magnitudes higher than the intrinsic absorption loss of a-Si. As shown in Figure S3a, when k increases, the broadening of the resonant bandwidth for quasi-BICs is not apparent but renders a shallower resonant depth in the spectra. In addition, the influence of k also depends on the intrinsic property of the excited quasi-BIC modes. For example, a broadening and shallower resonant profile is observed when k = 0.001 for the sample with Δx = -15 nm (Figure S3b) and takes places until k reaches 0.01 for the sample with Δx = -45 nm (Figure S3c). This is related to the fact that the magnitude of the induced near-field intensity drops significantly for kite-shaped nanopillars with a larger asymmetry level $|\Delta x|$ (Figure 2a). Thus, the non-radiative loss due to fabrication imperfection may not be the main mechanism for the broadening of quasi-BIC modes in our fabricated samples.

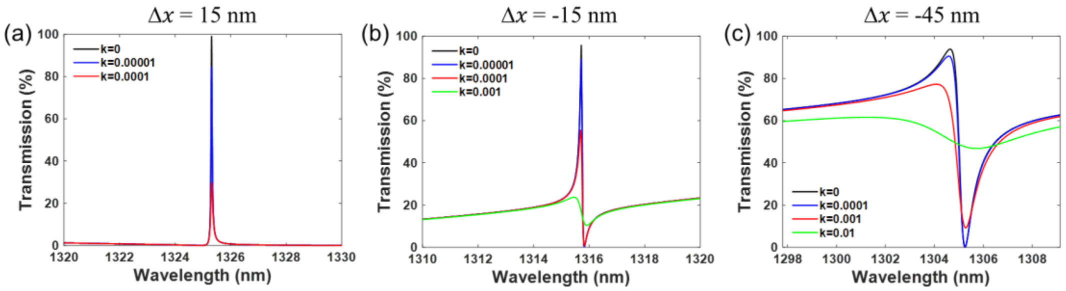

Figure S3. Simulated transmission spectra under different extinction coefficients of a-Si for kite-shaped metasurfaces with different asymmetry level of (a) Δx = 15 nm, (b) Δx = -15 nm, and (c) Δx = -45 nm.

Section S7: Effects of oblique incident angles on quasi-BICs

We used an in-house built microscope equipped with a 10× Infinity Corrected objective (a numerical aperture of 0.25) to measure the transmission spectra. The angular spread of the objective lens is estimated to be 1 to 14 degrees. To discuss how this angular spread affects the measured spectra of quasi-BICs, we first simulated the spectra of kite-shaped nanopillars at different incident angles (0-14 degrees). As displayed in Figures S4a to c, the quasi-BICs perform a spectral redshift along with a predominant broadening of the resonant linewidth at larger oblique incident angle. In addition, the amount of spectral broadening at oblique incidence is much larger for asymmetric nanokites with a larger cross-sectional area. We then modeled the measured response as a linear combination of different incident angles, and the contributions

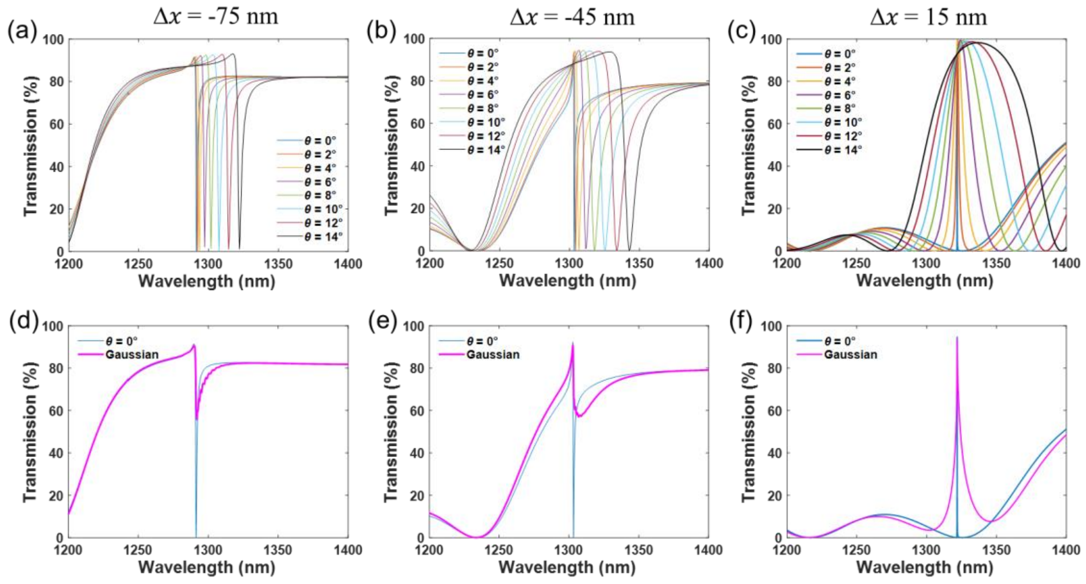

Figure S4. Simulated transmission spectra for kite-shaped nanopillars with an asymmetry level of (a) Δx = -75 nm, (b) Δx = -45 nm, and (c) Δx = 15 nm at different incident oblique angles. To estimate the effect of the unavoidable angular spread for the objective lens used in the measurement, we modeled the response by a linear combination of the spectra under different incident angles for nanokites with (d) Δx = -75 nm, (e) Δx = -45 nm, and (f) Δx = 15 nm. The blue curve refers the simulated spectra under normal incidence.

from each incident angle were estimated by a Gaussian distribution. As shown in Figures S3d to f, the calculated Gaussian-corrected spectra have a broadening linewidth with a shallower resonant depth, showing good correspondence to the measured spectra displayed in Figure 3b in the main text.

Section S8: Nonlinear numerical model for the THG response of all-dielectric metasurfaces

In order to make a more profound theoretical prediction for the effect of the quasi-BIC modes in boosting up the nonlinear conversion, a two-step nonlinear numerical model based on a perturbation theory was implemented to calculate the THG signals [3]. First, the electric field at the fundamental wavelength (FW) was calculated by the linear electrodynamic simulations. Then, the obtained electric field was employed to calculate the nonlinear polarization current as a new source for the nonlinear model through the relation of $P_i(3\omega) = \sum_{j,k,l} \chi^{(3)}_{ijkl} E_j(\omega)\, E_k(\omega)\, E_l(\omega)$, where $E(\omega)$ refer to the electric field at the FW, $P(3\omega)$ is nonlinear polarization at the TH wavelength, and $\chi^{(3)}$ is the third-order susceptibility. The value of $\chi^{(3)}$ is implemented as $2.45 \times 10^{-19}\, m^2/V^2$ for a-Si, and we only considered the dominant elements of $\chi_{xxxx} = \chi_{xxzz} = \chi_{xxyy} = \chi_{yyyy} = \chi_{yyzz} = \chi_{yxxy} = \chi_{zzzz} = \chi_{zyyz} = \chi_{zxxz}$ due to the cubic crystallographic point group of a-Si [4]. By solving the electrodynamic model at the TH frequency again, we can obtain the nonlinear response of dielectric metasurfaces. The THG signal was then evaluated by integrating the calculated THG electric field over the whole nanocavity volume (i.e. $I_{THG} \propto \int |E(3\omega)|^2 dv$).

Section S9: THG enhancement for nanokites with different asymmetry levels

The THG enhancement, defined as the measured THG intensity normalized by the THG signal obtained from the un-patterned Si film, are summarized in Table 1 for a series of nanokites with different asymmetry levels. The THG enhancement for these samples displays a similar trend to the measured THG intensity (without the normalization to the un-patterned Si film) shown in Figure 4d. The sample with Δx = 15 nm reaches a maximal THG enhancement beyond 2900-fold, and the THG enhancement degrades for samples with larger asymmetry levels.

Table 1. THG enhancement for nanokites with different asymmetry levels

Sample	THG enhancement
Δx = -75 nm	320
Δx = -45 nm	2593
Δx = -15 nm	2670
Δx = 15 nm	2962
Δx = 45 nm	833

References.

1. V. Savinov, V. Fedotov, and N. I. Zheludev, "Toroidal dipolar excitation and macroscopic electromagnetic properties of metamaterials," Physical Review B **89**, 205112 (2014).
2. A.-Y. Liu, J.-C. Hsieh, K.-I. Lin, S. H. Tseng, and H.-H. Hsiao, "Third Harmonic Generation Enhanced by Generalized Kerker Condition in All-Dielectric Metasurfaces," Advanced Optical Materials **11**, 2300526 (2023).
3. C. Ciracì, E. Poutrina, M. Scalora, and D. R. Smith, "Origin of second-harmonic generation enhancement in optical split-ring resonators," Physical Review B **85**, 201403 (2012).
4. J. E. Sipe, D. J. Moss, and H. M. van Driel, "Phenomenological theory of optical second- and third-harmonic generation from cubic centrosymmetric crystals," Physical Review B **35**, 1129-1141 (1987).
